# Dorsal Striatum Is Compromised by Status Epilepticus Induced in Immature Developing Animal Experimental Model of Mesial Temporal Lobe Epilepsy

**DOI:** 10.3390/ijms26073349

**Published:** 2025-04-03

**Authors:** Azzat Al-Redouan, Aaron Busch, Martin Salaj, Hana Kubova, Rastislav Druga

**Affiliations:** 1 Department of Anatomy, Second Medical Faculty, Charles University, 15006 Prague, Czech Republic; aaronbusch97@gmail.com (A.B.); martin.salaj@lfmotol.cuni.cz (M.S.); rastislav.druga@lf1.cuni.cz (R.D.); 2 Laboratory of Developmental Epileptology, Institute of Physiology, Czech Academy of Sciences, 14200 Prague, Czech Republic; hana.kubova@fgu.cas.cz; 3 Institute of Anatomy, First Faculty of Medicine, Charles University, 12000 Prague, Czech Republic

**Keywords:** basal ganglia, dorsal striatum, status epilepticus, epilepsy, seizure, degenerative neuronal changes, rat brain

## Abstract

This study investigated the striatopallidal complex’s involvement in status epilepticus (SE) caused by morphological neurodegenerative changes in a post-natal immature developing brain in a lithium−pilocarpine male *Wistar albino* rat model of mesial temporal lobe epilepsy. One hundred experimental pups were grouped by age as follows: 12, 15, 18, 21, and 25 days. SE was induced by lithium−pilocarpine. Brain sections were microscopically examined by Fluoro-Jade B fluorescence stain at intervals of 4, 12, 24, and 48 h and 1 week after SE. Each interval was composed of four induced SE pups and a control. Fluoro-Jade B positive neurons in the dorsal striatum (DS) were screened and plotted on stereotaxic rat brain maps. The DS showed consistent neuronal damage in pups aged 18, 21, and 25 days. The peak of the detected damage was observed in pups aged 18 days, and the start of the morphological sequela was observed 12 h post SE. The neuronal damage in the DS was distributed around its periphery, extending medially. The damaged neurons showed intense Fluoro-Jade B staining at the intervals of 12 and 24 h post SE. SE neuronal damage was evidenced in the post-natal developing brain selectively in the DS and was age-dependent with differing morphological sequela.

## 1. Introduction

Experimental studies indicate that status epilepticus (SE) causes neurodegenerative changes in the limbic structures (hippocampus, amygdala, parahippocampal cortices, piriform cortex, and claustrum), thalamus, and neocortical areas [1,2,3,4,5,6,7,8]. However, basal ganglia (BG) were rarely mentioned. Several clinical studies have reported brain abnormalities of atrophy and metabolic changes in structures remote from the seizure focus as well as in the BG [9]. Changes in the striatum, namely in the caudate nucleus, were detected and described in patients treated for epilepsy [10,11]. There are abundant experimental data, described through various experimental models of epilepsy, on degenerative changes in the hippocampus and related cortical areas and, similarly, in subcortical structures such as the amygdala, claustrum, and thalamus. However, such data concerning the striatopallidal complex (SPC) and related structures are sparse. There are significant data on neuronal damage in all analyzed structures in adult animals. Meanwhile, neuronal damage in the BG of developing brains in young epileptic animals is less reported [6,12,13,14,15].

### 1.1. Basal Ganglia Anatomy and Function

The brain structures that are included in the BG consist of the SPC, the subthalamic nucleus, and the substantia nigra (SN). The SPC constitutes a major integrative system of the forebrain, involved in the adaptive control of behavior and in the suppression of unwanted behavioral activities [16,17,18]. The SPC represents major subcortical accumulation of inhibitory (GABAergic) neurons and striatal efferents as well as efferent fibers emanating from both pallidal segments and substantia nigra (pars reticulata) [19,20,21,22]. The striatum, the globus pallidus (GP), the subthalamic nucleus, and the SN are considered to be the main components of the BG. The striatum is further subdivided into the dorsal striatum (DS) and ventral striatum (VS). The pallidal complex consists of external (GPe) and internal segments (GPi or entopeduncular nucleus EP). The SN is divided into the pars compacta (SNc) and the pars reticulata (SNr). The DS receives massive topographic glutamatergic projections from most parts of the cerebral cortex, and it receives additional projections from several thalamic nuclei, the pedunculopontine nucleus, and from the dopaminergic neurons of the SN [23,24,25]. The BG play an important role in decision making and the selection of context -appropriate actions. In rodents, the dorsolateral part of the striatum is involved in the control of innate and learned movements (habits), whereas the dorsomedial part is involved in novel movements. Another aspect of functional organization indicates that the dorsolateral striatum and its inputs are involved in motor control [26,27], while its ventromedial part is involved in limbic functions [28,29].

#### 1.1.1. Basal Ganglia Connectivity 

The characteristic connectivity of the BG are feed-forward projections from the cortex to the input nuclei (striatum and subthalamic nucleus), from the striatum and subthalamic nucleus to the GPe, and from these nuclei to the output structures (GPi and SNr). Output structures project to the thalamus [30,31,32]. These pathways form a partially closed loop named “the circuit of basal ganglia”, whose output link to the thalamus was discovered by Nauta and Mehler (1966) [33]. The striatum is the main input structure of the SPC. The entire cerebral cortex, several thalamic nuclei, and the amygdala project to the striatum [23,24,34,35,36,37]. All of these striatal afferents are glutamatergic and excitatory [18,19,29,38]. The transmission of cortical, thalamic, and amygdalar information through the striatum is modulated by dopaminergic and serotoninergic inputs from the SNc and the mesencephalic raphe nuclei [31,39,40]. The striatum projects to the GP and to the SNr [41]. The main output of the SPC is derived from the GPi and the SNr. These structures predominantly project to the thalamic nuclei and consequently influence the cerebral cortex [31,33,41]. All aforementioned efferent striatal projections are GABAergic and inhibitory [20,41].

The SPC is thus involved in a number of parallel functionally segregated cortico-subcortical circuits. These transthalamic circuits are sometimes designated as the “circuits of the BG” and represent a substrate for sensorimotor, cognitive, and motivational brain functions [18,21,33,42].

#### 1.1.2. Basal Ganglia Microstructure

The striatum is topographically organized into two histochemically defined parcellation compartments termed the striosomes (patches) and matrix. The striosomes form a three-dimensional labyrinth which occupy 10–15% of the striatal volume [43,44]. The extrastriosomal matrix is enriched by several neuropeptides and cholinergic markers. Both striatal compartments are differently involved in pathological processes affecting the striatum [45].

The striatum contains several cell types that are divided into two groups: projection neurons and interneurons [21,43,46]. Projection neurons are the most common cells in the striatum. In rodents, they represent about 95% of neurons and are GABAergic and divided into two subpopulations. One expresses substance P and dopamine 1 receptor; their axons target the output nuclei (GPi and SNr) and represent the “direct pathway” which can initiate movements. The other subpopulation expresses enkephalin and dopamine 2 receptors; their axons project to the GPe and represent the “indirect pathway” with an inhibitory effect on the BG output nuclei [16,41]. These are medium-sized ovoid cells (14–20 µm), and their dendrites heavily protrude spines [32,42,47,48,49]. The predominant striatal element is a medium-sized, densely spiny neuron. The remaining neurons are short axonal local inhibitory interneurons. One population of interneurons expresses acetylcholine, while the remaining ones express GABAergic interneurons, which colocalize calcium-binding proteins (parvalbumin and calretinin), neuropeptides (neuropeptide Y and somatostatin), and NOS (NADPH-d) [32,38].

### 1.2. Seizures and Status Epilepticus

Status epilepticus is defined as a period of seizure activity lasting for at least 30 min during which full consciousness does not recover [50]. The latent period is defined as the time between the initial brain insult and the clinical manifestation of the first seizure. This seizure-free period can last for many years in humans, but it is in the range of days to weeks in animal model experimentations [51,52,53,54]. During the latent period, there are alterations in neuronal and glial cell structure and function despite the asymptomatic clinical presentation [4,55].

### 1.3. Current Clinical and Experimental Data Concerning the Basal Ganglia in EPILEPSY

Several clinical and experimental studies have indicated that SPC cannot generate epileptic seizures and are unlikely to be involved in their initiation [56]. However, these clinical and experimental studies have also suggested that the BG may be part of a control system modulating the spread of epileptic seizures [9,14,15,40,56,57,58]. Structural damage in epileptic patients and in experimental animals is extensive and is not limited to structures of the limbic system [4,6,59,60]. Imaging techniques, such as MRI, fMR, PET, and SPECT, revealed metabolic involvement of the striatum during seizure activity, causing atrophy in the caudate nucleus [4,9,11,14,40,60,61]. Various SE mature rat experimental models demonstrated neurodegenerative changes in the striatum, the activation of neurons (c-Fos expression), differential changes in calcium-binding proteins, and changes in glucose metabolism and in the density of GABAergic neurons [12,41,61,62,63,64,65,66]. Pilocarpine-induced SE led to a significant decrease in striatum acetylcholine activity [66,67,68]. Covolán and Mello (2000) [12] described a difference in the regional distribution of striatal neuronal damage between kainate and pilocarpine models. However, detailed topography of epileptic neuronal damage within the SPC and other components of the BG of developing brains in young immature animals is lacking in the literature. Our long-term aim is to investigate the potential SE-caused neurodegenerative changes in structures that are traditionally regarded as neither being associated with the generation nor propagation of epileptic seizures.

### 1.4. Purpose and Objectives

As stated above, clinical as well as experimental studies have demonstrated that SE damage is more severe in the mature brain [13,15]. However, much less is known about the immature developing brain in comparison to the adult brain. Neuronal degeneration may also have different localization and course in the immature brain [13,67,69]. In this study, we investigated neuronal damage in the BG in experimental animal models with an age interval of 12 to 25 post-natal days, which corresponds to the toddlers’ population in humans who are 1 to 3 years old [52]. Morphological manifestation of acute short-term to long-term sequelae was also considered by setting the post SE intervals at four hours to one week. These intervals are comparable to the duration of approximately five days to eight months in humans, as one day in a laboratory rat’s life cycle equals approximately one month (34.8 days) in a human’s life cycle according to Sengupta (2013) [52]. The experiment was performed on lithium–pilocarpine-induced SE rats, which represent models of mesial temporal lobe epilepsy (MTLE) [53,70,71].

## 2. Results

**Figure 1:**
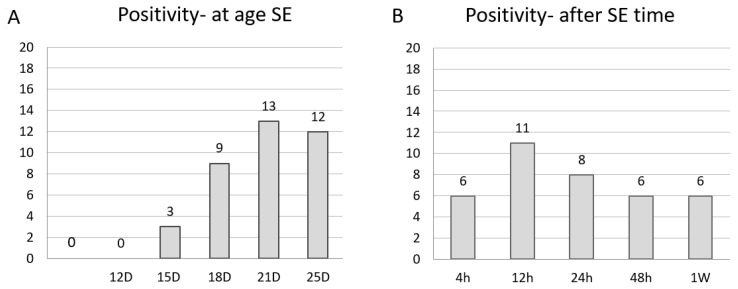
A graphical representation of the total number of experimental animals exhibiting FJB-positive neurons, comparing its incidence as follows: (**A**) the age of SE onset, indicating higher FJB-positive incidence at an age of 21 days; (**B**) after the SE time interval, indicating higher FJB-positive incidence in the survival interval of 12 h after SE. n = 100 experimental (25 control). Legend: SE—status epilepticus; D—days; h—hours.

**Figure 2:**
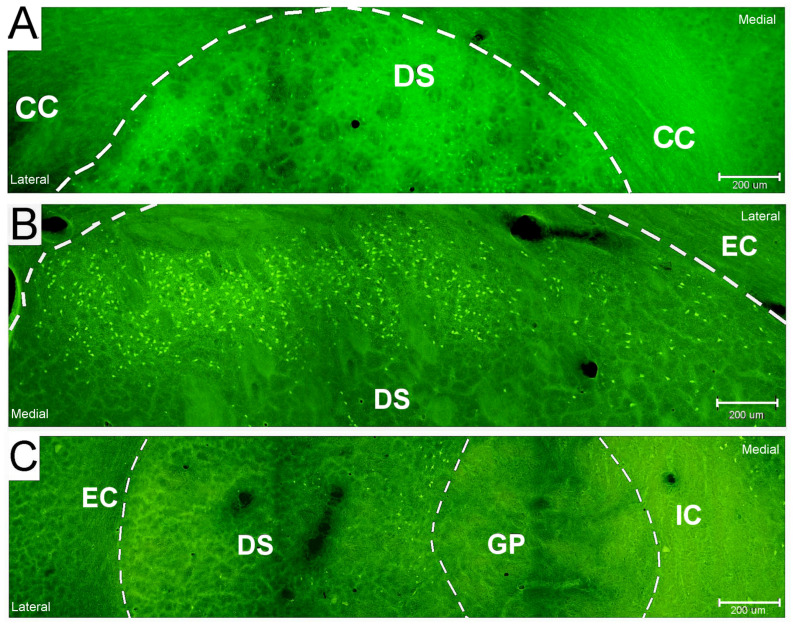
Microscopic photographs showing the distribution of Fluoro-Jade B-positive neurons detected within the periphery in the dorsal striatum of the rat brain sections (magnification—20×). (**A**) At bregma 2.28, showing the rostral sector of the DS (age of 21 days, survival interval of 1 W). (**B**). At bregma 0, showing the middle sector of the DS (age of 21 days, survival interval of 24 h). (**C**) At bregma −2.16, showing the caudal sector of the DS (age of 21 days, survival interval of 12 h). Each figure is a construction of three visual field photos auto-aligned using CellF Software, Olympus (version 2.8). Legend: DS—dorsal striatum; GP – globus pallidus; CC—corpus callosum; EC—external capsule; IC—internal capsule.

**Figure 3:**
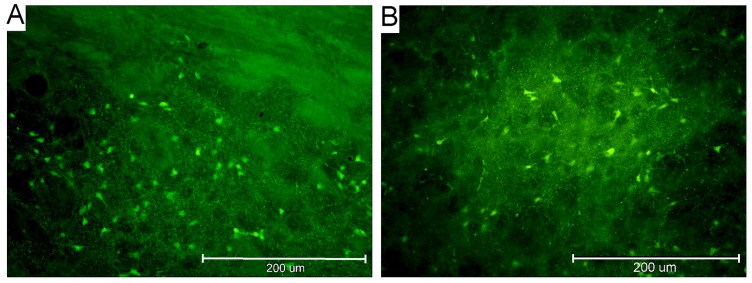
Microscopic photographs showing the damaged Fluoro-Jade B-positive neurons in the dorsal striatum of the rat brain sections (magnification—40×). (**A**) At the age of 25 days, 12 h post SE, exhibiting intense staining of cell bodies. (**B**) At the age of 25 days, 1 week post SE, exhibiting some shrunken positive neurons with less stain intensity surrounded by a “dusty” background. Legend: SE—status epilepticus.

**Figure 4:**
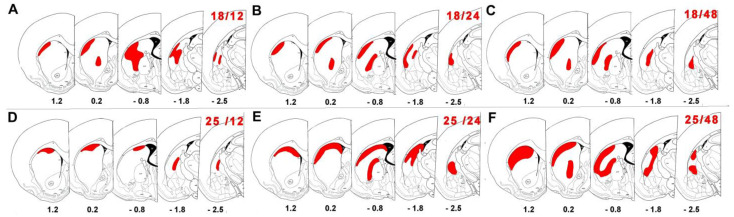
Schematic representation of coronal brain sections demonstrating distribution of FJD-B-positive cells in dorsal striatum of pups that experienced status epilepticus. (**A**) At age of 18 days, 12 h post SE. (**B**) At age of 18 days, 24 h post SE. (**C**) At age of 18 days, 48 h post SE. (**D**) At age of 25 days, 12 h post SE. (**E**) At age of 25 days, 24 h post SE. (**F**) At age of 25 days, 48 h post SE. Legend: SE—status epilepticus. The number in the upper left corner of each figure refers to age/survival interval. Number below each figure refers to distance from bregma [72]. Schemes were constructed using CorelDraw Software (version 11).

The detected positivity in the BG structures is reported in Table 1. The total number of animals exhibiting FJB-positive neurons is illustrated graphically by age and survival intervals in Figure 1, showing that more pups (65% incidence) aged 21 days developed SE, and it was more prominent with an incidence of 55% at a survival interval of 12 h after SE. In P18, P21, and P25 rats, DS neuronal damage was consistently observed (Figure 2) at all post SE time intervals with the exception of P18 at 4 h (Table 1). FJB-positive neurons exhibited intense staining of the cell body at intervals of up to 24 h after SE, while some of the positive neurons were shrunken, less intensely stained, and surrounded by a “dusty” background at longer survival intervals (48 h and 1 W) (Figure 3). Figure 4 illustrates the overall observed distribution of neuronal damage in the DS, and it is described in more detail below. 

### 2.1. Age-Specific Pattern

#### 2.1.1. In P12

There were no degenerated (FJB-positive) neurons in all survival intervals (Figure 1) (Table 1).

#### 2.1.2. In P15

Only isolated degenerated (FJB-positive) neurons were found at the survival intervals of 24 h and 48 h after SE in the rostral half of the DS. Degenerated neurons prevailed in the dorsal marginal part of the striatum. There were no FJB-positive neurons in the GP, the subthalamic nucleus, or the SN (Table 1).

#### 2.1.3. In P18

A small number of degenerating neurons were evident in the rostral half of the DS along its dorsolateral margin in the shorter survival intervals (8 h and 12 h). There were degenerated (FJB-positive) neurons dispersed in the central part of the caudal half of the DS. A moderate number of degenerated (FJB-positive) neurons were found concentrated along the dorsolateral margin within the rostral half of the DS, and there was another area of neuronal degeneration found more caudally discernible in the medial part of the DS adjacent to the GP in the longer survival intervals (24 h and 48 h) (Figure 2 and Figure 4). The GP, subthalamic nucleus, and SN did not contain any FJB-positive neurons (Table 1).

#### 2.1.4. In P21 and P25 

The distribution of degenerating neurons (FJB-positive) in the DS was very similar in these two age groups. The severity of striatal damage reached a peak at 24–48 h after SE in these two groups of animals. However, the distribution of degenerated neurons remained unchanged one week after SE with a noticeable decrease in the density of FJB-positive cells. The degenerated neurons (FJB-positive) were concentrated along the dorsolateral margin of the rostral half of the DS in survival intervals of 12 h–48 h. In addition, another area of neurodegeneration was evident caudally in the DS lateral to the GP. Through the caudal sections, degenerated neurons (FJB-positive) prevailed in the basal part of the DS extending to the vicinity of the amygdalar complex (Figure 4). Similarly, to the other age groups, the GP, subthalamic nucleus, and SN did not contain any FJB-positive neurons (Table 1). 

### 2.2. Neuronal Damage Distribution

**Figure 5:**
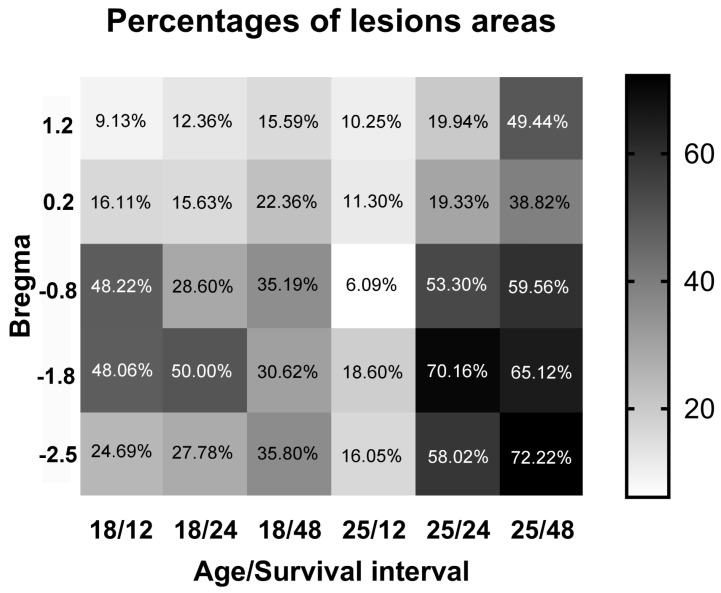
Heat map illustrating percentages of area occupied by lesion within selected intervals of rat brain’s bregma in FJB-positive neurons in 18- and 25-day-old pups at survival intervals of 12 h, 24 h, and 48 h, corresponding to destruction illustrated in Figure 4, indicating differing pattern of density by age and survival intervals based on bregma level.

The detected degenerated neurons formed a continuous marginal belt within the dorsolateral aspect of the DS without a distinct fluctuation in the density of FJB-positive neurons in all age and survival interval groups. An analogous distribution of degenerating neurons was also evident in the peri-pallidal part of the DS (Figure 2 and Figure 4). The extent of the damaged neuronal cluster measured by the percentage area within the DS varied by age and survival interval at different levels of the brain’s bregma (Figure 5). There was a gradual increase in neuronal damage to the nucleus area ratio further dorsally in the FJB-positive brains. The damaged area increased in ratio at the middle bregma of P18 at survival intervals of 12 h and 24 h but remained large at the survival interval of 48 h to its dorsal bregma. However, the compromised area ratio was constant in P25 at the survival interval of 12 h, indicating less spread of neuronal damage in this specific group. On the other hand, the compromised area showed wider spread with a gradual increase from the anterior at bregma 1.2 to the posterior at bregma −2.5 in P25 at the survival intervals of 24 h and 48 h (Figure 5).

### 2.3. Characteristics of FJB-Positive Neurons

**Figure 6:**
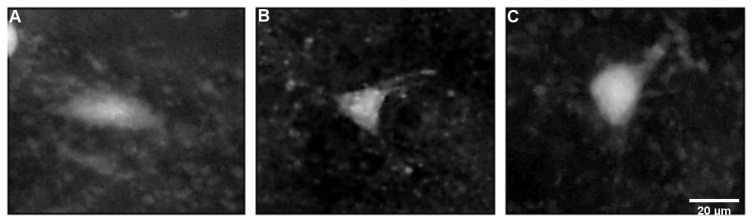
Fluoroscopic photomicrographs showing degenerated neuron shapes detected by Fluoro-Jade B in dorsal striatum in 18-, 21-, and 25-day-old pups at survival interval of 24 h after status epilepticus. Magnification = 45×. (**A**) Spindle. (**B**) Triangular. (**C**) Oval.

**Figure 7:**
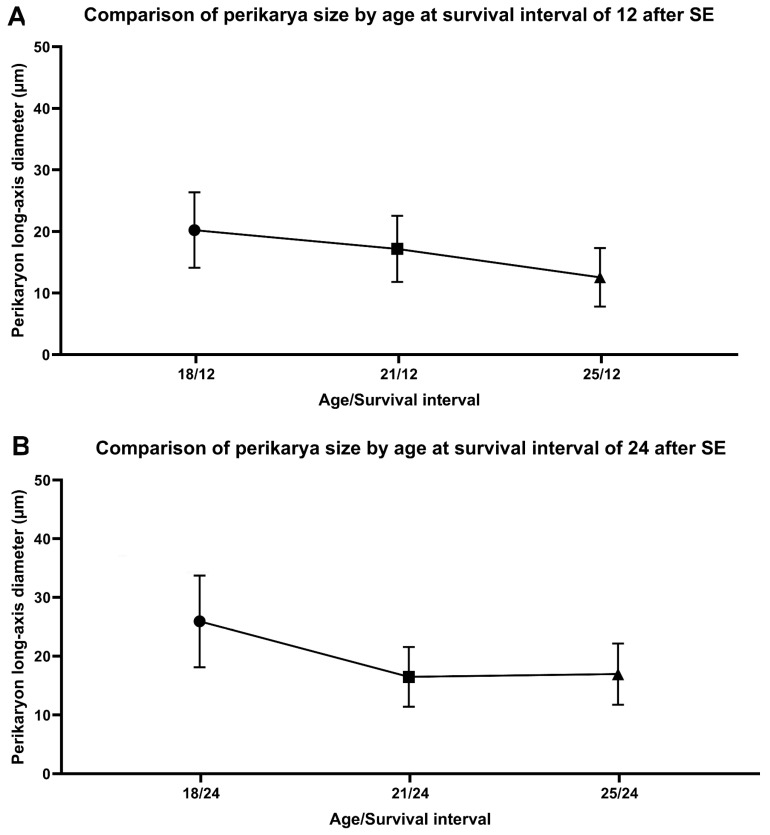
Graphical presentation illustrating changes in size of FJB-positive perikarya in age groups of 18, 21, and 25 days. (**A**) At survival interval of 12 h after SE indicating a decrease in size by age intervals. (**B**) At survival interval of 24 h after SE. Legend: SE—status epilepticus indicating decrease in size between ages of 18 and 21 days.

The FJB-positive neurons exhibited intense staining of the cell body at intervals of up to 24 h after SE (Figure 2A). Meanwhile, some of the positive neurons were shrunken, less intensely stained, and surrounded by a “dusty” background at longer survival intervals of 48 h and 1 week (Figure 2B). This indicates a disintegration of dendritic processes after the SE time interval. The FJB-positive neurons in the P18 pups showed a more disguisable shape than in older pups, namely those in the P21 and P25 groups. The FJB-positive neurons showed three distinctive shapes: spindle, triangular, and oval (Figure 6). The size of the perikarya (long-axis diameter) was smaller at the SE survival interval of 12 h in P18 and P25 (Table 2). There was evidence of a decrease in size (long-axis diameter) by age in the survival interval of 12 h after SE (Figure 7A). The size (Table 2) decreased between P18 and the other two age groups of P21 and P25 in both survival intervals of 12 h and 24 h (*p*-value < 0.05) but remained at a similar size between P21 and P25 in the survival interval of 24 h after SE (Figure 7B).

### 2.4. Control Animals

There were no FJB-positive neurons in the DS nor in the other analyzed structures in all of the control animals.

## 3. Discussion

The MTLE arises in the hippocampus and adjacent structures, causing prominent neuronal damage in the amygdala [73]. Our SE animal experimental observation confirms remote expression in the BG in the pups aged 18 days and above. Existing studies [58] in the literature addressed the involvement of the BG in temporal lobe epilepsies in animal studies [5,14,57] as well as in clinical studies [45]. Epilepsy is a common neurological disorder that occurs more frequently in children than in adults. The circuit of the BG is thought to be a part of a control system inhibiting the spread of epileptic seizures in several models of epilepsy [40,54]. Our present results provide, for the first time, evidence that lithium/pilocarpine-induced SE produces neuronal degeneration in the DS in immature rats, and its severity of damage is highly related to age and survival intervals. While neuronal degeneration was sparse in the P15 rats by means of quantitative degenerated neurons, it increased in the older animals (P18, P21, and P25).

### 3.1. Age-Specific Pattern of Neuronal Damage in Status Epilepticus

The peak of neuronal damage incidence by SE at post-natal age was observed in our study in P18, and the peak after SE based on the survival time interval was observed 12 h post SE (Figure 4). This demonstrates evidence of age-dependent neuronal damage in the DS. A previous study also demonstrated post SE age-dependent damage in the thalamus of lithium–pilocarpine experimental pups with similar age intervals [4,74]. Overall, the DS at the younger age of P15 seemed to be more resilient. One possible explanation for such an observed phenomenon is the different features of SE during developmental stages of differing maturity of the synaptic contacts, namely in glutamatergic synapses [67]. The density of asymmetric but not symmetric synapses increases after P15. Spine density in medium spiny neurons increases drastically between the ages of 15 and 20 days in rats [49]. 

The long-term post SE damage (one week in pups is equivalent to more than six months in human toddlers) showed severe DS neuron morphological distortion (Figure 3). Several studies elaborated on the long-term effect of seizures [3,55,59,75]. Doczi et al. (2003) [3] illustrated late-term electrophysiological sequela in developing brains of induced epileptic rats. A clinical study by Scott et al. (2014) [59] suggested long-term permanent damage in repetitive exposure to MTLE and SE in humans. 

### 3.2. Neuronal Damage Distribution

The damaged neurons detected in the DS were distributed in a distinctive pattern and tended to cluster around the peripheries with medial expansion of neuronal damage over time after SE (Figure 4). This territorial confined manifestation could correlate with the afferent striatal projections in mature adult animals. Degenerating neurons in the rostral half of the DS overlap significantly with the corticostriatal projections from the SI area [76], the posterior parietal cortex [77], the orbital area (VLO), the MI area, the premotor area, and the visual association area (Oc2M) [36]. Degenerating neurons in the caudal half of the DS starting approximately at bregma −0.60 overlap significantly with the corticostriatal projections from the auditory area [78] and the amygdalostriatal inhibitory projections in the basolateral nucleus [34,39]. The presence of degenerated axonal fibers within intra-striatal fasciculi indicates damage to striatal projecting neurons and corticofugal fibers. Hyperactivity of corticostriatal and amygdalostriatal glutamatergic projections after SE together with the post-natal development of striatal synapses may result in excessive glutamate release and thus in the development of excitotoxic damage in the striatal neurons [10,49,57,67], as detailed in the below section.

### 3.3. Mechanisms of Neuronal Damage Observed in Status Epilepticus

One possible mechanism of striatal damage following lithium–pilocarpine-induced SE is glutamate-induced neurotoxicity. Rapid neuronal death induced by glutamate excitotoxicity is regarded as the initial phase of a seizure-induced effect in the brain [79]. The main striatal afferent systems originating in the neocortex, thalamus, and amygdala operate with excitatory amino acid transmission [31,38]. Afferent striatal projections were analyzed predominantly in adult rats. Among the afferent striatal systems, the corticostriatal system represents dominant input with synaptic contacts on the projecting GABAergic neurons and on the parvalbumin-immunoreactive striatal interneurons [32,41]. The topography of striatal damage corresponds to termination areas of the corticostriatal systems that originate from different neocortical areas. This, in turn, indicates a correlated involvement in the neuronal damage caused by SE. The dorsolateral area of the rostral half of the DS contained moderate to massive neurodegeneration, which roughly corresponds to the terminal striatal projections of the barrel cortex, the agranular medial and lateral cortex, and the occipital area Oc2M [36,37,43]. Striatal projections from these areas significantly overlap within the dorsolateral marginal part of the DS where, in our experiment, the degenerated striatal neurons caused by SE were distributed. The dorsolateral focus of neuronal damage is also partly overlapped with the projections from the vibrissal representations in the primary motor and the somatosensory cortical area, with the projections arising from the hindlimb and forelimb somatosensory cortical areas, and with the projections from intralaminar thalamic nuclei (paracentral and centralis lateralis nuclei) [31,80]. In contrast to the projections from the medial orbital cortex, the ventral and the ventrolateral orbital cortices terminate in the medial and central striatal regions [81]. The dorsolateral part of the DS is also targeted by projections from the lateral posterior thalamic nucleus [82]. Thus, the convergence of several cortical and subcortical (excitatory) projections to the dorsolateral part of the DS probably caused distinct neuronal degeneration. Projections from the agranular cortex and from the vibrissal and limb cortical areas terminate in the calbindin-poor somatomotor within the DS [83]. A focus of degenerated neurons in the posterior half of the DS adjoining to the GP and basally to the amygdalar complex partly overlap with the projections from the dorsal agranular insular area; from the ventrolateral, lateral, and dorsolateral orbital cortices; and from the amygdala [34,35,36,73,81]. The question of whether degenerated neurons are located within the matrix or the striosomes was answered by demonstrating the corticostriatal innervation of both striatal compartments at least from cingulate and agranular cortices by Kincaid and Wilson (1996) [43]. Therefore, the hyperactivities in these overlapping striatopetal systems may result in excessive glutamate release and lead to the development of excitotoxic damage, as originally described by Turski et al. (1984) [84] and later summarized by Holopainen (2008) [79].

Less severe SE-induced neuronal degeneration in the DS in P15 animals may be related to the maturation of the principal striatal spiny neurons and their synaptic contacts. It is known that the majority of asymmetric (excitatory) synaptic contacts are not yet mature in very young animals’ synaptic apparatus, and they are formed during the third post-natal week [49]. This is in agreement with our results. Distinct and more extensive neuronal damage in the DS was observed for the first time in P18.

Degenerating neurons never occurred in the GP, EP, nor in the SN. Resistance of these structures is difficult to explain but may be influenced by specific patterns of input and a high concentration of inhibitory neurons. Resistance in the GP and in the SN may be related to the paucity of glutamatergic neocortical projections and the high concentration of GABAergic and parvalbumin-immunoreactive neurons [85]. It should be emphasized that hyperactivities of striatal projecting neurons may stimulate inhibitory mechanisms within both segments of the GP and in the SNr. The subthalamic nucleus is under the influence of excitatory neocortical projection and comprises a large amount of GABAergic projection from the GPe, which may also ensure an inhibitory and protecting interference. In addition, the subthalamic nucleus is densely populated by parvalbumin-immunoreactive interneurons [49,86,87].

### 3.4. BG Neuronal Degeneration in Other Status Epilepticus Experimental Models 

Data from previously published studies on SE-induced neuronal activation and damage in the SPC are not consistent and are influenced by the ages of animals and by the experimental model of epilepsy [12]. Microinjection of the cholinergic agonist carbachol into the thalamic convulsive seizure area (reticular thalamic nucleus and VPL nucleus) resulted in epileptic seizures and in a distinct increase in Fos-positive neurons for up to 2 h after SE in the DS with a small increased compromised area in the EP and in the SN [62]. However, a detailed description of its distribution and the activated neurons in the BG were not addressed. 

Generalized tonic–clonic seizures in Noda epileptic rats failed to increase Fos expression in the SPC [64,65,78]. In contrast to this, a significant increase in Fos expression was observed in the cerebral cortex and in several limbic structures [65]. Seizures induced by pilocarpine in immature rats (P21) resulted in the downregulation of muscarinic and dopaminergic receptors and in decreases in dopamine and serotonin levels [67]. Nevertheless, our results also demonstrate resistance in several nuclei within the circuit of the BG.

### 3.5. The Limitations of This Study

As a limitation of the used method, no specific effort was made to conduct an investigation of the relation of FJB-positive neurons to the matrix and striosome compartments of the DS. The distribution of FJB-labeled neurons suggests that the degenerated neurons are dispersed mainly in the matrix, but neuronal degeneration within the striosomes cannot be excluded.

## 4. Materials and Methods

This was a semi-quantitative observational animal model experimentation performed in accordance with a protocol used in previous studies conducted by Druga et al. (2005) [6] and Al-Redouan et al. (2024) [88] with modifications to the experimental design, while animal handling and the invasive procedure used were in accordance with the ethical policy.

### 4.1. Experimental Animal Model and Design

A total of 125 male *Wistar albino* rats were divided into five groups as follows: 12 (P12), 15 (P15), 18 (P18), 21 (P21), and 25 (P25) post-natal days. Each age group consisted of 20 experimental subjects and 5 control subjects. Status epileptic seizure was induced by lithium−pilocarpine injection in the 20 experimental animals (100 in total) in each group, and then the animals were sacrificed and prepared for morphological analysis at intervals of 4 hours (4 h), 12 hours (12 h), 24 hours (24 h), 48 hours (48 h), and 1 week (1 W). Each survival interval was composed of 4 experimental animals and 1 control animal (Table 1). The manifested motor activity of seizure (facial muscles twitching, jaw clenching, head nodding, erected tail, forelimb clonus, and shuffling movements) were monitored, and to standardize the experimental groups, animals with secondarily generalized tonic–clonic seizures were excluded from this experiment. 

The brains of the animals experienced SE, and the control animals were sectioned into 50 μm coronal sections and stained with Fluoro-Jade B (FJD-B) histochemistry according to Schmued and Hopkins (2000) [89,90] because dying neurons provide an FJD-B-positive fluorescence signal [89,90] (Figure 1). The brain sections were independently evaluated microscopically (Olympus Bx51 FITC filter fluorescence microscope equipped with Olympus DP72 digital camera with installed QuickPHOTOMicro 2.3 software) by two observers (blind control for reliability measure). FJD-B positivity was screened in the BG by observing the dorsal striatum (DS), globus pallidus (GP), and entopeduncular nucleus (EP) (Table 1). Anatomical localization and bordering of these BG were aided by 1-in-5 adjunct Nissl (cresyl violet)-stained brain sections. The DS was observed sequentially on cross-sections from bregma 1.2 to −2.5, where it is bordered by corpus callosum superiorly, external capsule laterally, and lateral ventricle along with internal capsule and GP medially. It was separated from the VS by a vertical line running between the end of the external capsule to the lateral ventricle. The detected FJD-B-positive neurons were plotted on schemes of brain sections corresponding to the stereotactic brain map by Paxinos and Watson (2007) [72]. This was accomplished by first placing a 250 um by 250 um square grid covering the DS area followed by superimposing the grid squares containing more than ten FJD-B-positive neurons on scanned plots using CorelDraw software, Alludo, Ottawa, ON, Canada (version 11).

#### 4.1.1. Brain Handing and Histological Preparation

Rats were euthanized as described below in Section 4.4. Then, the brains were fixed for three hours after being removed from the rats’ skulls and cryoprotected in graded sucrose of 10%, 20%, and 30% in PBS. Afterward, the brains were frozen in dry ice and stored at −70 °C. 

Serial sections of 1 in 5 were mounted on gelatin-coated slides and processed for FJB histochemistry according to Schmued and Hopkins (2000) [89,90]. These brain sections were examined with an epifluorescence microscope using fluorescein thiocyanate filter sets.

#### 4.1.2. Statistical Analysis 

The *t*-test was used to evaluate the statistical significance in terms of *p*-values set at *p* < 0.05 between the compared groups of differing age and survival intervals. After that, a two-way ANOVA was employed to estimate the means of the quantitative variables (Supplementary File S1).

### 4.2. Sample Size Statistical Calculation

The effective sample size (E) was determined based on a crude method and evaluated using the “resource equation”, where E should be more than 20 and not less than 10 [91,92].

E = (Number of animals per group × Number of groups) − Total number of groups [93]

This calculation was applied on both the overall grouping by age (A) and the sub-grouping by pos-SE interval (B) (Table 1).

(A) Five groups of 20 per group: E = (20 × 5) − 5 = 95 → (>20)

(B) Five groups of 4 per group: E = (4 × 5) − 5 = 15 → (lies between 10 and 20)

### 4.3. Animal Handling and Ethical Policy of Invasive Procedures

Animal care and experimental procedures were conducted in accordance with the guidelines of the European Community Council directives 86/609 EEC. Experiments were approved by the Animal Care and Use Committee of the Institute of Physiology of the Academy of Sciences of the Czech Republic. The study and access to specimens were approved for research and education purposes by the Institutional Review Board (IRB)—The Ethics Committee of the University Hospital Motol and Second Faculty of Medicine, Charles University, Prague, Czech Republic [reference ID no. EK-1175.1.19/22].

### 4.4. The Procedure and Administered Drugs

SE was induced by lithium–pilocarpine intraperitoneal injections. First, lithium chloride (LiCl) (3 mmol/mL/kg, i.p.) was administered, and 24 h later, pilocarpine (40 mg/mL/kg i.p.) was administered. Two hours after the onset of SE, motor seizures were suppressed by intraperitoneal injection of paraldehyde (0.3 mL/kg for P12, P15, and P18 rats, while 0.6 mL/kg was used for P21 and P25 animals, i.p.) (no. 76260, Fluka Chemie AG, Buchs, Switzerland). Control animals were treated with equal volumes of LiCl, but the pilocarpine was replaced with saline followed by the corresponding paraldehyde dose with the same timing as their counterpart groups. Animals were deeply anesthetized with urethane (2.5 g/kg, i.p) and perfused with 0.01 M sodium phosphate-buffered saline (pH 7.4, room temperature) followed by 4% paraformaldehyde in 0.1 M phosphate buffer (pH 7.4 (1 mL/g), +4 °C).

## 5. Conclusions

The DS showed consistent neuronal damage in pups aged 18, 21, and 25 days. The peak of the detected damage was observed in pups aged 18 days, and the start of the morphological sequela was observed 12 h post SE. Neuronal damage in the DS was distributed around its periphery, extending medially. The damaged neurons showed intense Fluoro-Jade B staining at intervals of 12 and 24 h post SE, while a lower staining intensity with a dusty background was observed at the intervals of 48 h and 1 week. SE neuronal damage was evidenced in the BG of the post-natal developing brain selectively in the DS and was age-dependent with differing morphological sequela. 

## Figures and Tables

**Table 1:** Fluoro-Jade B positive and negative findings indicating neuronal damage by animal age and post status epilepticus survival intervals.

Age at SE	12 D			15 D			18 D			21 D			25 D		
Basal Ganglia	DS	GP	EP	DS	GP	EP	DS	GP	EP	DS	GP	EP	DS	GP	EP
Survival Interval															
4 h	Negative	Negative	Negative	Negative	Negative	Negative	Negative	Negative	Negative	Positive	Negative	Negative	Positive	Negative	Negative
12 h	Negative	Negative	Negative	Negative	Negative	Negative	Positive	Negative	Negative	Positive	Negative	Negative	Positive	Negative	Negative
24 h	Negative	Negative	Negative	Positive	Negative	Negative	Positive	Negative	Negative	Positive	Negative	Negative	Positive	Negative	Negative
48 h	Negative	Negative	Negative	Positive	Negative	Negative	Positive	Negative	Negative	Positive	Negative	Negative	Positive	Negative	Negative
1 W	Negative	Negative	Negative	Negative	Negative	Negative	Positive	Negative	Negative	Positive	Negative	Negative	Positive	Negative	Negative

Age intervals are indicated by days (12, 15, 18, 21, and 25 days), and survival intervals by hours (4, 12, 24, and 48 h) and 1 week. n = 100 (75 experiment + 25 control). Legend: DS—dorsal striatum; GP—globus pallidus; EP—entopeduncular nucleus.

**Table 2:** Average perikaryon long-axis perimeter (µm) in Fluoro-Jade B-positive neurons by age and survival intervals where perikaryon was not disintegrated with visible cell shape.

Age/Survival Interval	18/12	18/24	21/12	21/24	25/12	25/24	*p*-Value
Perimeter (µm)	20	26	16	17	13	17	
SD	±3.28	±3.22	±3.00	±3.00	±3.30	±2.79	*p* < 0.05

## Data Availability

The supporting data are attached as a Supplementary Materials.

## Supplementary Materials

The following supporting information can be downloaded at: https://www.mdpi.com/article/10.3390/ijms26073349/s1.

## Abbreviations

SE	status epilepticus
BG	basal ganglia
DS	dorsal striatum
GP	globus pallidus
GPe	globus pallidus external segment
GPi	globus pallidus internal segments
SNc	substantia nigra pars compacta
SNr	substantia nigra pars reticulata
